# Intestinal perforation due to colorectal cancer during pregnancy: case report and literature review

**DOI:** 10.1186/s12884-024-06533-9

**Published:** 2024-05-16

**Authors:** Yan Gao, Yu Sun

**Affiliations:** Department of Obstetrics and Gynecology, Peking University First Hospital, Beijing, China

**Keywords:** Colorectal cancer, Pregnancy, Intestinal perforation, Intestinal obstruction

## Abstract

Colorectal cancer (CRC) in pregnancy is sporadic. We reported a case of a woman at 23 + 4 weeks of gestation who presented with abdominal pain. The patient underwent an ultrasound and MRI, during which a colonic mass was noted. Considering a probable incomplete intestinal obstruction, a colonoscopy, biopsy, and colonic stenting were performed by a multidisciplinary team. However, sudden hyperthermia and CT demonstrated intestinal perforation, and an emergency caesarean section and colostomy were conducted. The histological analysis confirmed moderately high-grade adenocarcinoma.

## Case presentation

A 36-year-old Chinese woman (gravida 0 para 0) with no family history of cancer presented at the Emergency Department of Peking University First Hospital, Beijing, China, at 23 ^+4^ weeks of pregnancy. She complained of 3-day diarrhoea and aggregating abdominal pain. The patient had iron deficiency anaemia for five years and rectal bleeding for two years, which was treated with sigmoidoscopy and haemorrhoid surgery, and she denied other diseases in her remote pathological history.

**Fig. 1:**
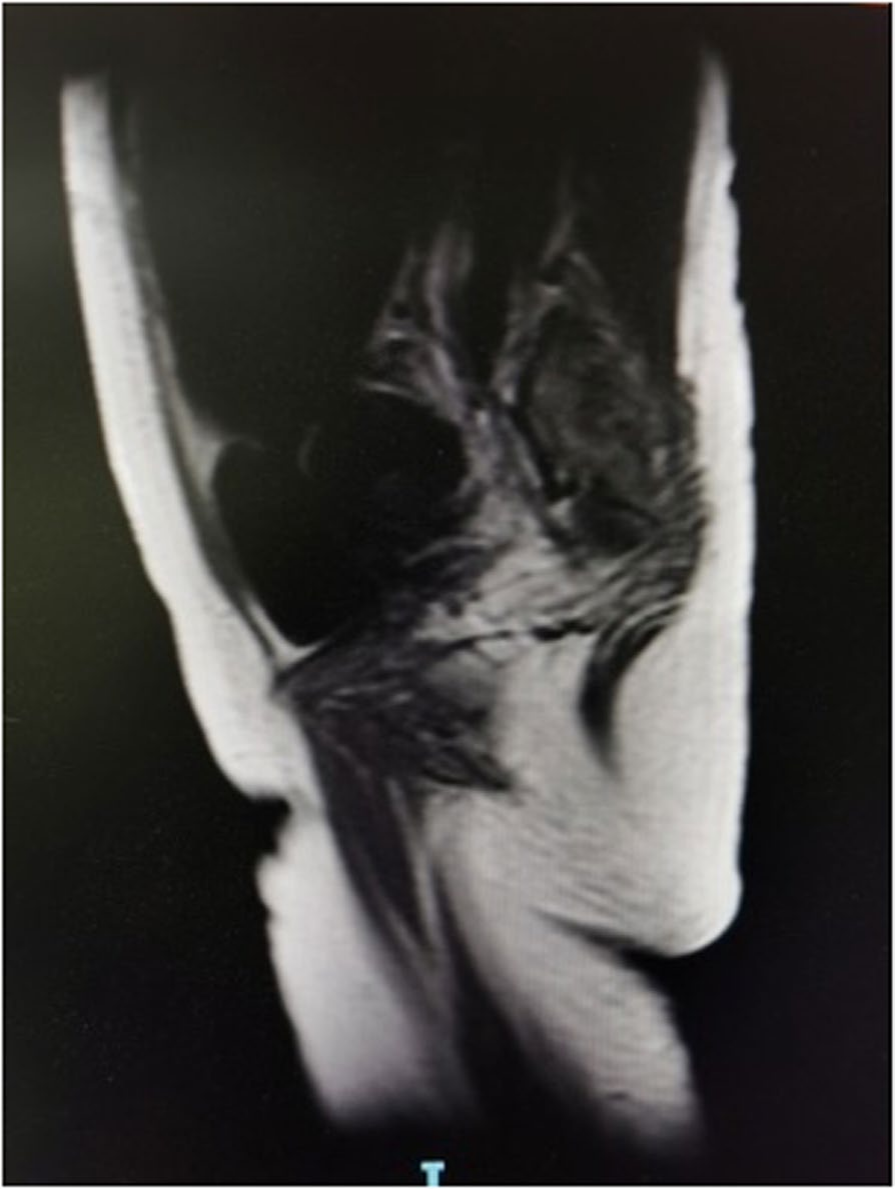
MRI at admission

Upon admission, physical examination revealed abdominal tenderness and rebound pain, particularly in the right upper quadrant (RUQ) and below the xiphoid. There was a suspicious tenderness at McBurney's point. The laboratory examination showed as follows: white blood cell (WBC) count 10.51X10^9^/L, haemoglobin (Hb) 82 g/L, C-reactive protein (CRP) 40 mg/L, potassium 3.08 mmol/L, sodium 131.21mmo1/L; otherwise, the coagulation function, amylase, lipase, and liver and kidney function were within normal range. Abdominal ultrasound demonstrated dilated intestines at the left upper quadrant and a mass measuring 8 cm behind the uterus suggestive of intestinal origin. The abdominal and pelvic Magnetic Resonance Imaging (MRI) without contrast indicated an irregular thickening of the large bowel between the colon and sigmoid colon, with an extension of 8-10 cm (Fig. 1). Therefore, a rectum and sigmoid colon neoplasm accompanied by incomplete intestinal obstruction was the initial suspicion.

Considering the complexity of the patient's condition, a multidisciplinary meeting was organised, including the department of obstetrics, general surgery, haematology, endoscopy, imaging department, and other departments. During the discussion, all the participating departments agreed with the initial suspicion of colon cancer (T4aN + Mx). A colonoscopy was recommended to clarify the diagnosis further and to decide whether termination of pregnancy was appropriate based on the pathological results.

**Fig. 2:**
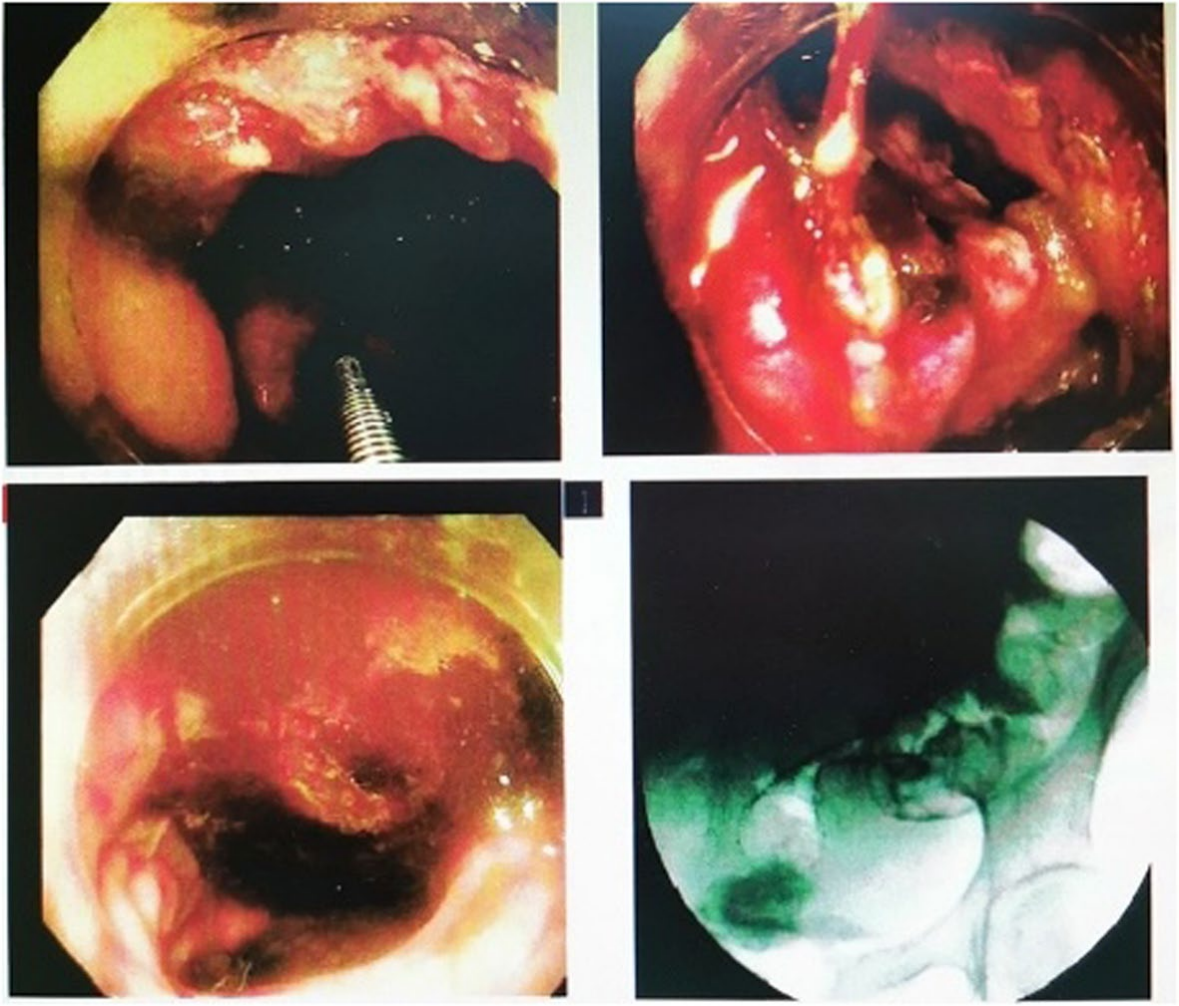
Colonoscopy findings

An emergency sedation-free colonoscopy was performed and revealed an exophytic circumferential mass located at the junction of the rectum and sigmoid colon, 13 cm away from the anus. The mass surface was ulcerated and covered with white moss. Additionally, there was a narrowing lumen of 3-4 cm in length, which did not allow for the endoscope to progress (Fig. 2). The biopsy was taken. Meantime, anti-infection therapy, iron intravenous iron supplementation therapy, and parenteral nutrition were initiated.

On the second day after admission, the patient's pain was relieved. The stool was soft and yellow, with a small amount of red mucus visible on the surface. Since the haemoglobin level gradually decreased to 69 g/L, 2 Units of red blood cells were transfused to correct anaemia. After a blood transfusion, the patient developed a fever, with a maximum temperature of 38.9 ℃, accompanied by chills, shortness of breath, and wheezing (50–60 breaths/minute). Blood oxygen saturation was 91% and 94% after oxygen uptake of 3L/min, blood pressure was 102/60 mmHg. Full blood count (FBC) showed WBC 10.07 × 10^9^/L, Hb 81 g/L, platelet 204 × 10^9^/L, and CRP 146 mg/L. Blood gas analysis showed a pH of 7.46, an oxygen saturation index of 94%, and oxygen partial pressure of 65 mmHg. An emergent computed tomography (CT) suggested intestinal perforation due to colon lesions.

Consequently, at 23^+5^ weeks of gestation, an emergency cesarean section and transverse colostomy were performed simultaneously with the collaboration of obstetricians and a colorectal surgeon. A transverse incision was made, and a large lesion occupying the sigmoid colon was detected during the operation, surrounded by enlarged lymph nodes. No metastatic nodules were found in the peritoneum, omentum, and pelvic wall, and no apparent signs of perforation were observed in the intestinal canal above the retroflexion of the peritoneum. A latex drainage tube behind the uterus was placed. After the surgery, the patient was transferred to the Surgical Intensive Care Unit (SICU), where she received treatment including acid suppression, anti-infection therapy and parenteral nutritional support, which gradually transitioned to a regular diet. Eventually, the patient was discharged on the 11th day post-operation.

The postoperative histopathological result demonstrated moderately to high differentiated adenocarcinoma. Immunohistochemical staining results were listed: P53 90% + , Her-20, MLH1 + , PMS2 + , MSH2 + , MSH6 + . Microsatellite stability: MSS. No tumour cells were found in the placenta. Under the guidance of general surgery, the patient took capecitabine orally 25 days after surgery and planned to undergo two courses of intravenous chemotherapy before undergoing further surgical treatment.

## Discussion

Colorectal cancer (CRC) in pregnancy is exceedingly rare. The incidence rate of CRC in pregnancy is 0.002% to 0.008% [1]. Typical symptoms/signs include hematochezia or melena, abdominal pain, apart from unexplained iron deficiency anaemia, or a change in bowel habits [2]. Less common presenting symptoms include abdominal distention or nausea and vomiting, which may be indicators of obstruction.

A delayed diagnosis during pregnancy may be due to overlapping symptoms with that of normal pregnancy in the context of the expected low incidence of CRC at such an early maternal age. The reluctance of medical teams to conduct diagnostic tests due to potential risks to the fetus often leads to a delayed diagnosis, thus complicating treatment and worsening the prognosis [3]. Various serum markers have been associated with CRC, particularly carcinoembryonic antigen (CEA). However, all these markers, including CEA, have a low diagnostic ability to detect primary CRC due to significant overlap with benign disease and low sensitivity for early-stage disease [4].

Nonionising radiation imaging, such as Ultrasound and MRI, is favoured during pregnancy. Despite low intrauterine doses, CT scan can be used in pregnancy if necessary.

Diagnostic imaging studies typically expose the fetus to less than 50 mGy (0.05 Gy, five rads), and there is no evidence of an increased risk of fetal anomalies, intellectual disability, growth restriction, or pregnancy loss from ionising radiation at this dose level [5, 6].

In the United States and elsewhere, the standard practice at most institutions is that all patients with stage II, III, or IV CRC undergo chest, abdomen, and pelvic CT before or after resection, an approach endorsed by the National Comprehensive Cancer Network.

Colonoscopy is the most accurate diagnostic test for CRC since it can localise and biopsy lesions throughout the large bowel, detect synchronous neoplasms and remove polyps. Endoscopy is recommended in pregnancy when the patient has significant or continuous bleeding, severe or refractory nausea and vomiting or abdominal pain, and strong suspicion of colon mass [7].

The optimal time for advanced endoscopic procedures during the pregnancy is the second trimester; however, if the consequences of a delayed procedure can cause harm to the patient or the fetus, then one should proceed with a multidisciplinary team [8].

GI endoscopy in pregnant patients is inherently risky because the fetus is susceptible to maternal hypoxia and hypotension, either of which could lead to fetal demise. Other risks to the fetus include teratogenesis (from medications given to the mother or ionising radiation exposure) and premature birth [9].

A systematic review was conducted to identify studies regarding CRC-p and conduct a pooled analysis of the reported data. Seventy-nine papers written on 119 patients with unequivocal CRC-p were included. The calculated pooled risk is 0.002%, and age at diagnosis has decreased over time. The median age at diagnosis was 32 (range, 17–46) years. 12%, 41% and 47% of CRC-p were diagnosed during the first, second and third trimester respectively. Among the cases, bleeding occurred in 47% of patients, abdominal pain in 37.6%, constipation in 14.1%, obstruction in 9.4% and perforation in 2.4%. Regarding cancer, 53.4% of the CRC-p was in the colon, while 44% was in the rectum. Out of 82 patients whose treatment was described, 9.8% received chemotherapy during pregnancy. None of their newborns developed permanent disability, one developed hypothyroidism, and 72% of newborns were alive. Hence, treatment of CRC-p should be timely and needs to be discussed carefully by a multidisciplinary team, with close patient monitoring [10].

Treatment of CRC-p is influenced by several factors, including tumor location and stage at presentation, surgical settings (elective vs. urgent/ emergent) and gestational age. The decision-making process must involve anesthesiologists, colorectal surgeons, oncologists and gynaecologists, while the mother will make the final decision.

If the tumour is resectable, surgical excision after diagnosis should be performed as soon as possible if the diagnosis is made before 20 weeks of gestation. A total abdominal hysterectomy may be necessary to provide greater access to the rectum or if the uterus is involved. If diagnosis is made later in pregnancy (> 20 weeks), surgery can be postponed until fetal pulmonary maturity is reached (28–32 weeks) or after delivery. However, waiting until after the fetus is delivered does pose risks to the mother, and the patient should be fully informed of these risks [11].

When malignant neoplasm is diagnosed during gestation, maternal life-saving chemotherapy poses life-threatening concerns for the developing fetus [12]. Exposure to chemotherapy in the first trimester poses the most significant risk for teratogenicity, with an incidence of spontaneous abortions or malformations up to 15–25% [13]. In the second or third trimesters, chemotherapy is generally considered safer but is associated with an increased incidence of small for gestational age fetuses (SGA) [14, 15]. Chemotherapy is often continued until 35 gestational weeks or three weeks before the expected due date. Timing is recommended to avoid the increased risk of chemotherapy-related complications, such as bone marrow suppression, bleeding, and maternal and fetal death during delivery [16].

A distance of > 30 cm from the field edges will expose the embryo/fetus to only 4–20 cGy. Therefore, many areas (e.g., head and neck, breast, and extremities) can be treated with radiation. Lead shielding over the embryo or fetus can also reduce the exposure. Because of the location of the tumour and proximity of the embryo/fetus, Radiation therapy (RT) is contraindicated during pregnancy. Radiation therapy can be used postoperatively only after delivery or elective abortion in pregnant patients. Future fertility should be considered before treatment because RT can cause permanent damage to the ovaries, which can lead to infertility [11, 17].

Colorectal cancer is the most common cause of large bowel obstruction, comprising 60 per cent of all cases.

Patients who present with an acute malignant colorectal obstruction may require immediate surgery if they have a perforation or pending perforation, or if they are clinically unstable (tachycardic, hypotensive, acidotic), or are symptomatic. Perforation occurs more commonly at the point of obstruction, most likely due to local tumour invasion or inflammatory reaction, rather than in the proximal, dilated colon. The decision to choose a staged versus one-stage procedure depends upon several factors, including the location of the obstructing lesion, condition of the proximal colon, medical comorbidities of the patient, as well as their life expectancy, goals of care, and the presence of proximal perforation [18–20].

## Conclusion

Colorectal cancer in pregnancy is rare. For patients whose symptoms include chronic anaemia that is difficult to correct with iron supplements and persistent gastrointestinal symptoms during pregnancy, it is necessary to be vigilant about the possibility of digestive tract tumours. It's essential to note that tumours should be suspected when all benign causes are excluded despite low incidence [21, 22]. Ultrasound and magnetic resonance imaging are relatively safe examinations during pregnancy, and if necessary, pelvic CT and gastroscopy can be performed for diagnosis.

## Data Availability

All data generated or analysed during this study are included in this published article.
